# Examining the Range and Scope of Artists’ Professional Practices With Individuals With Palliative Care Needs: An International, Cross-Sectional Online Survey

**DOI:** 10.3389/fpsyg.2021.773451

**Published:** 2021-12-09

**Authors:** Jenny Baxley Lee, Sonja McIlfatrick, Lisa Fitzpatrick

**Affiliations:** ^1^Institute of Nursing and Health Sciences Research, Ulster University, Coleraine, Northern Ireland; ^2^Center for Arts in Medicine, College of the Arts, University of Florida, Gainesville, FL, United States; ^3^School of Arts and Humanities, Ulster University, Coleraine, Northern Ireland

**Keywords:** artists, arts in health, palliative care, end-of-life care, patients, cross-sectional survey

## Abstract

**Background:** Internationally, it is recognized that artists facilitate arts engagement with individuals with palliative care needs. There is a gap in the literature describing the range and scope of artists’ professional practices in palliative care. The aim of this study was to examine an international range of professional practices among artists who work in palliative care including key professionals’ perceptions of these practices.

**Methods:** An international, cross-sectional, online survey was conducted with health professionals, artists, and program coordinators with experience with artists working in palliative care. This survey was part of a larger mixed methods study. An instrument was systematically developed to examine artists’ professional practices. Descriptive statistics were reported for the total sample including frequencies, means and standard deviations and open-ended items were analyzed thematically.

**Results:** 101 valid surveys were analyzed. Findings outlined: (1) who delivers the arts; (2) where and with whom; (3) practice descriptors; and (4) perceptions of practice. Themes identified from open-ended items on benefits and risks of practice revealed impacts on patients and artists alike, including: (1) enhanced well-being; (2) vulnerabilities; and (3) facilitators and barriers.

**Conclusion:** Findings demonstrated a wide range of artists’ practices in palliative and end-of-life care, featuring notable consistencies in international practice worth further exploration. Ongoing and international efforts examining artists’ practices in palliative care contribute to the development of future research, policy and practice.

## Introduction

It is recognized that artists facilitate arts engagement with individuals with palliative care needs in a range of settings such as hospitals and hospice (Anderson et al., 2017; Peng et al., 2019; Lee et al., 2021). Individuals with palliative care needs experience distinct impacts to their quality of life and well-being as daily routines, social connection, and a sense of identity are significantly disrupted (Sepúlveda et al., 2002; Long, 2011; Knaul et al., 2018; Greer et al., 2020; Henson et al., 2020). In response, palliative care offers a range of modalities to address quality of life through life-affirming care with an emphasis on social support and a team approach (Meier and Brawley, 2011; World Health Organization, 2018). Evidence points to the benefits of supportive modalities such as the arts to address non-pharmacological symptom management, quality of life and meaning making (Charmaz, 2006; Long, 2011; Moss and O’Neill, 2014; Wilson et al., 2016; Peng et al., 2019). Arts engagement aligns with palliative care aims by engaging creativity and expression thereby enhancing well-being through discovery, agency, meaning, connection with others, and a sense of self beyond illness (Kennett, 2000; Charmaz, 2006; Long, 2011; Anderson et al., 2017; Peng et al., 2019). A vast body of evidence supports arts engagement for health and well-being, broadly (Staricoff, 2004; Macnaughton et al., 2005; Clift, 2012; Fancourt et al., 2014; Fancourt and Finn, 2019). A gap exists, however, in the evidence describing the range and scope of artists’ professional practices in palliative and end-of-life care.

Much of the evidence underpinning artists’ work in palliative care is drawn from broad health contexts such as bedside practice in hospitals or from creative arts therapies’ literature (Moss and O’Neill, 2009). Field-wide definitions of arts in health aim to distinguish the professional roles of artists from creative and expressive arts therapists as drawn from the National Organization for Arts in Health’s white paper (National Organization for Arts in Health, 2017), and yet these distinctions in role and scope frequently blur in practice. In this paper, the term arts in palliative care is inclusive of visual, literary, performing, and multi-disciplinary arts engagement provided by an artists with individuals with palliative care needs. Arts in palliative care is contextualized within the broader discipline of arts in health, which includes arts engagement delivered within a healthcare or clinical context, also commonly referred to as arts in medicine or arts in healthcare (Macnaughton et al., 2005; National Organization for Arts in Health, 2017; Sonke et al., 2018).

Evidence demonstrates the importance of skilled practice of artists working in healthcare (Moss and O’Neill, 2009, 2019; Sonke, 2015; Moss, 2016; Tan, 2018, 2020; Van Lith and Spooner, 2018). Delineation between the professional roles and practices of artists and creative arts therapists in palliative care serves to direct the scope of practice, education, and training needs of each (Moss and O’Neill, 2009; Van Lith and Spooner, 2018). Artists delivering the arts in healthcare settings must be skilled and knowledgeable to safely and effectively navigate within healthcare environments including following infection control protocols, documentation of practice, and interprofessional communication, for example (Moss and O’Neill, 2009; Jensen, 2014; Sonke, 2015). Tan (2018, 2020) offers a conceptual model for the practice of a “caring artist” in the context of a nursing home, pointing to the artist’s capacity to attune, assess, and adjust with consideration for participant well-being, the environment, and quality arts activities. Assessment and observation, project planning, collaboration, individual and group facilitation, research, and reflexivity were identified as key components of professional practice among artists working in healthcare (Moss and O’Neill, 2009; Jensen, 2014; Tan, 2018, 2020; Van Lith and Spooner, 2018). As arts in health is not widely formalized or systematically available, communication skills and a common vernacular are also invaluable in order to translate the values and aims of arts engagement with patients for effective interprofessional collaboration (Jensen, 2014; Sonke et al., 2017, 2019). Despite this growing body of literature, there are few published studies to date describing the mechanisms and nuances of professional practice among artists working in palliative or end-of-life care settings (Tan, 2020; Lee et al., 2021).

A lack of evidence exists to describe artists’ professional pathways into palliative care including training, preparation or resources to safeguard their well-being and resilience as a workforce (Moss and O’Neill, 2009; Van Lith and Spooner, 2018). This is a critical consideration and protective factor for any healthcare-based workforce (Mills et al., 2020). Therefore, there is a significant need for further conceptualization of professional practices of artists working in palliative care. This paper presents a critical examination of the international range and variation with artists’ practice in palliative care, as called for in the extant literature (Raw et al., 2012; Jensen, 2014; Tan, 2020). Critical analysis of patterns of consistency and variation in artists’ practice alongside facilitators and barriers reported by key professionals in arts in palliative care steers a more nuanced conceptualization of practice in an effort to inform future research, policy, and practice toward greater efforts to increase uptake of the arts in palliative care.

The aim of this study was to examine an international range of professional practices among artists who work in palliative care including key professionals’ perceptions of these practices.

For the purpose of this study, key professionals included health professionals, artists, and program coordinators with direct experience with artists working with individuals with palliative or end-of-life care needs.

## Materials and Methods

An international, cross-sectional online survey was conducted to describe and examine key professionals’ perceptions of the range and scope of artists’ practices including education and training, art forms, settings and participant populations, and approaches to practice. This survey was part of a larger convergent parallel mixed methods study. It is underpinned by pragmatism in its intent to emphasize complementarity of quantitative and qualitative approaches toward shared meaning and joint action (Morgan, 2007, 2014; Shannon-Baker, 2016; Moseholm and Fetters, 2017). A 27-item survey instrument was designed in English using Qualtrics, a secure web-based survey platform. The survey was provided in English as this is a common language utilized by the identified international arts in health professional membership organizations through whom the survey was distributed. Ethical approvals and oversight were provided by the institutional review boards of both affiliate institutions (RG-0119 and IRB 201902947). A participant information sheet was provided at the outset of the survey and respondents provided informed consent in the survey itself prior to commencing completion of the instrument. Survey methods and findings were reported following Eysenbach’s Checklist for Reporting Findings of Internet Surveys (CHERRIES; Eysenbach, 2004).

### Instrument Design and Development

As there was no existing validated survey instrument designed to systematically detail artists’ practice in healthcare generally, nor in palliative care specifically, an instrument was developed. This scoping survey was designed to examine the range of practice contexts, scope of practice, multiple key descriptors of levels of arts engagement, and professional structures such as the nature of professional collaboration, documentation of practice and treatment of publicly-engaged patient created art works by art form. The development of the survey instrument was iterative, inductive, and informed by both the literature and by conferring with subject-matter experts (Wilson et al., 2016; Eva and Morgan, 2018). Testing and piloting the instrument assisted with ensuring the clarity and sequencing of each item and the length of time to completion. The researcher (J.B.L.) consulted a statistician (J.M.) to ensure that the survey instrument was designed, and the quantitative data analyzed, in a systematic and rigorous manner.

The survey instrument included 27 items categorized by domain or topic and placed in a logical order. The first and second domains focused on range and scope of practice and included 14 closed-ended questions in which respondents selected from a pre-determined list from multiple choices or a ranking scale. The last domain included four open-ended questions on perceptions of practice. The survey instrument was divided into the following sections: (1) eligibility screening and demographics such as geographical location, employment, level of education and training specific to arts in health; (2) in-depth practice descriptors such as who delivers the arts, where and with whom, number of arts sessions facilitated per week with individuals or groups by health condition and art form as well as observable changes during and following arts engagement; and (3) key professionals’ perceptions of practice, including perceived benefits and risks of arts engagement in palliative care. See Table 1 for the survey instrument.

**TABLE 1:** Survey instrument.

1. [Screening] Have you engaged professionally with the arts in palliative care? Yes (1) No (2) 2. [Current professional role] Select one answer below that best describes your current professional role with the arts in palliative care. [Branching Logic-Change of role] If you have had a change of professional role, such as you are retired from practice, or are not currently working with this population, your work and knowledge is vital. Please describe your work in arts in palliative care below. 3. [Multiple roles] If you have had multiple professional roles related to the arts in palliative care, please describe in the space below. 4. [Demographics: Country of residence] In which country do you currently reside? 5. [Demographics: Education level] What is the highest level of school you have completed? 6. [Training in arts in health] Have you received training specific to delivering the arts in healthcare? Yes (1) No (2) 7. [Training in arts in health] Please briefly describe your training in delivering the arts in healthcare. 8. [Current place of employment] Which one of the following best describes your current place of employment? 9. [Years of employment] How many years have you been in your current professional role? 0–30 years slider 10. [Arts engagement by setting] In which of the following settings have you engaged with the arts? (Select all that apply.) 11. [Years of professional engagement in arts in palliative care] How many years have you been professionally involved with the arts in palliative care? 0–30 slider 12. [Artistic discipline/s] Which art forms are engaged with patients in your practice or program? Please specify in the space below. 13. [Profession of arts facilitator] Who delivers the arts with patients in your practice or program? Select all that apply. 14. [Professional collaborations to deliver the arts] Who collaborates professionally to deliver the arts in your practice or program? Select all that apply. 15. [Common health conditions among arts participants] What are some common health conditions among patients who participate? Select all that apply. 16. [# of sessions per week] Over the past year, what is the average number of arts sessions provided per week with patients in your practice or program? 17. [Average length of session] What is the average length of an arts session in your practice or program? 18. [Percentage of time spent by participant/group] Approximately what percentage of time is spent with individuals and groups in your practice or program? With individuals on a one-to-one basis: _______ (1) With groups of individuals who have a similar health condition: _______ (2) With groups of individuals with varying health conditions: _______ (3) With groups of individuals who have health conditions and their family members: _______ (4) With groups of individuals who have health conditions and healthcare professionals: _______ (5) Other – (please describe): _______ (8) Total: ________ 19. [Age range of participants] Approximately what percentage of patients in the following age ranges engage in the arts in your practice or program? 0–3 years of age: _______ (1) 4–12 years of age: _______ (2) 13–18 years of age: _______ (3) 19–35 years of age: _______ (4) 36–64 years of age: _______ (5) 65 years of age or older: _______ (6) Total: ________ 20. [Source of referrals] Approximately what percentage of the following provide patient referrals to the arts in your practice or program? Artist: _______ (8) Program coordinator: _______ (5) Family members or loved ones: _______ (2) Patient self-referral: _______ (1) Health professional – (please specify): _______ (3) Total: ________ 21. [Outcomes] What if any, changes do you observe during, or immediately following, an arts session in your practice or program? Select all that apply. Change observed in patient health or symptoms – please specify. (1) ________________________________________________ Change observed in patient’s environment such as lighting, sound, seating arrangement, etc. – please specify. (2) ________________________________________________ Change as verbalized by patient or group – please specify. (5) ________________________________________________ Other (6) ________________________________________________ 22. [Documentation of practices] Do artists (or those who deliver the arts) document their experience working with patients in your practice or program? Select all that apply. Artists document arts sessions in a medical record. (1) Artists document arts sessions to share with their supervisor and/or colleagues. (2) Artists document arts sessions for professional purposes such as record of time spent, grant reporting, annual reporting, etc. (3) Artists document arts sessions for research and/or evaluation purposes. (7) Artists document arts session for other purposes. – (please describe) (6) _____________ Artists do not formally document arts sessions. (4) 23. [Use of artworks] When a patient engages in an arts session with your program, is the artwork produced shared publicly in any form, such as an exhibit or performance? Yes – (please describe) (1) ______________________________________________ No – (please describe) (2) _______________________________________________ 24. [Meaning of palliative care in professional context] Briefly, what does the term “palliative care” mean to you in the context of your professional work? 25. [Benefits] In your view, what are the primary benefits, if any, of engaging the arts with patients in palliative care? 26. [Risks] In your view, what are the primary risks, if any, of engaging the arts with patients in palliative care? 27. [Open-ended] What else would you like to share about your professional experience engaging the arts in palliative care?W3510

### Inclusion Criteria

Inclusion criteria required respondents to have directly engaged professionally with the arts in palliative care as either an artist, a health professional, a program coordinator, or a researcher. As stated in the background, definitions were aligned with field publications in arts in health, which defines an artist working in healthcare as distinct from a credentialed creative arts therapist (Moss and O’Neill, 2009; Sonke, 2015; National Organization for Arts in Health, 2017; Sonke et al., 2018). The survey made this delineation explicit at the outset by clearly stating that creative arts therapists should participate in the survey in the role of *health professional* rather than *artist*. An open-ended text item invited respondents to expound on their role to account for professional role variation or multiple roles. The inclusion criterion, “direct professional experience working with artists delivering arts engagement” therefore included creative arts therapists who actively made patient referrals to, or otherwise collaborated with, artists in their respective palliative or end-of-life care programs.

### Sampling and Recruitment

A purposive sample of key professionals with direct experience with artists’ work with individuals with palliative care needs was invited to complete a securely hosted, online survey in Qualtrics based on their specialized knowledge of the subject matter (Kalton, 1983; Ruel et al., 2015). The study was not designed to achieve statistical significance or to test a statistically representative sample. No published data was identified that articulated the number of artists working in palliative care worldwide or by region, hence the rationale for the present study. It was therefore not possible to state the confidence level and margin of error. Further, membership of the identified arts in health networks and organizations represent professionals across sectors including practitioners, health professionals, program coordinators, researchers and commissioners associated with arts in health broadly, therefore an unknown percentage of members work in palliative care.

An initial recruitment strategy included an email invitation from within each organization to the members of three international arts in health professional membership organizations: the United States-based *National Organization for Arts in Health* (*n* = 339 members; 2,011 newsletter subscribers*)*; *Culture, Health and Well-being Alliance* in the United Kingdom (*n* = 4,395 members and newsletter subscribers); and *Arts and Health Australia* (*n* = *NA)*. The administrators of the *National Organization of Arts in Health* and the *Culture Health and Well-being Alliance* included the invitation in their respective e-newsletters and posted on social media platforms such as Twitter and Facebook. Respondents were encouraged to distribute the survey among their own networks. To further expand the reach, additional professional membership organizations such as the European Association for Palliative Care, Medical and Health Humanities Africa, the British Association for Art Therapists (BAAT) and the BAAT special interest group in palliative and cancer care distributed the survey through their memberships and networks *via* email and on social media.

### Data Collection

The online survey remained open from November 26, 2019 through January 15, 2020. Reminder emails and social media reminders were sent following initial invitations in weeks one, two, four and six to enhance response and the survey period was extended two weeks into January 2020 to account for the holiday period. The survey stated anticipated length of time to complete and aim of the study. Ethical considerations were provided in a linked participant information sheet including study aim and details, the voluntary nature of the study, potential risks and benefits, the data management plan, and study contacts. Informed consent was collected following the participant information sheet and prior to the eligibility screening question. No incentives were provided to survey respondents for their participation. To ensure that data remained anonymous, a separate, unlinked survey collected contact information of those respondents who wished to remain in contact for future study activities.

### Data Analysis

Data were analyzed by members of the research team (J.B.L. and J.M.) using descriptive statistical analysis and thematic analysis (Braun and Clarke, 2006, 2014) and informed by a pragmatic approach (Morgan, 2007, 2014;Creswell and Creswell, 2017; Miles et al., 2018). Initial reports of the raw data were reviewed using Qualtrics, a secure web-based survey management and analysis platform. SAS 9.4 software (SAS Institute Inc., Cary, NC, United States) was used to further analyze quantitative data and generate descriptive statistics to identify characteristics of the sample and inferential statistics to determine statistical significance. As the primary objective of this project was to describe the range and scope of artists’ practices with individuals with palliative care needs, descriptive statistics were reported for the total sample (frequencies, means and standard deviations). Beyond this, three variables were used for further stratification: current professional role (coded as artist, health professional, or program coordinator), country of residence (coded as United States versus non-United States), and current place of employment (coded as arts organization, hospital/inpatient, university, or other). For each stratified analysis, chi-square and Fisher’s exact test statistics were reported for categorical variables.

Qualitative responses to open-ended items were coded and thematic analysis conducted in MaxQDA using six stages identified by Braun and Clarke (2006; 2014): (1) data familiarization; (2) initial coding; (3) theming; (4) review themes; (5) define themes; and (6) writing. The first author undertook line-by-line open coding to develop an initial set of codes. Codes and sub-codes aimed to capture both semantic and conceptual or latent meaning (Braun and Clarke, 2006). The codes were then organized categorically and re-coded to develop primary and secondary categories from which a set of themes were developed by the principal investigator. Discussion and verification were then undertaken by the research team to review, refine, combine, or discard themes and to ensure they were firmly grounded in the data (Lincoln and Guba, 1985, 1986). Once verified, each theme was organized into a table to display data in relation to the research question in order to facilitate theme generation. Qualitative data introduced unique insights into the perceptions of respondents as reported and discussed below.

## Findings

A total of 151 responses were received. After cleaning the data, removing tests (4) and duplicates (6) and accounting for those with greater than 50% missing items (40), 101 valid survey responses were analyzed in full.

### Respondent Characteristics

Of the 101 surveys analyzed in full, forty-nine respondents identified willingness to participate in an interview in a subsequent study phase. For the purposes of the article, those who completed surveys reported herein were referenced as *respondents*. The term *participants* is reserved to identify individuals who participated in arts engagement such as patients, service users, or other individuals with palliative care needs.

**FIGURE 1:**
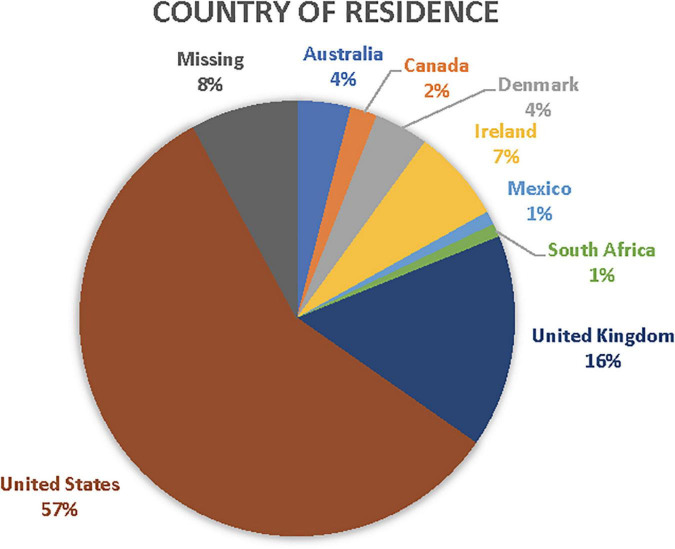
Respondents’ country of residence.

Among the survey respondents (*n* = 101), 38% identify as artists, 34% health professionals, 24% program coordinators, 4% researchers representing relatively equal distribution across roles. Geographically, 57% of respondents were US-based whilst 35% were non-US based including 16% from the United Kingdom, 7% from Ireland, 4% from Australia and Denmark, respectively, 2% from Canada, and less than 1% from Mexico and South Africa. As there were no forced responses to any survey item with the exception of consent and screening for inclusion, 8% of responses to country of residence were missing. See Figure 1 for survey respondents’ country of residence.

Regarding qualifications, a majority of those who responded held graduate or master’s degrees (47%) whilst 30% held undergraduate degrees (bachelor’s or associate’s) and 17% held doctoral or professional degrees such as a medical degree (MD). Approximately 67% had received training specific to arts in health practice. Respondent characteristics are detailed in Table 2.

**TABLE 2:** Respondent characteristics.

The count (n) and percentage (%) are reported as n (%), unless otherwise stated.	Total (*n* = 101)
**Primary professional role**	
Artist	38 (37.6)
Health professional	34 (33.7)
Program coordinator	24 (23.8)
Researcher	4 (4.0)
Change of professional role	1 (0.1)
**Country of residence**	
Australia	4 (4.0)
Canada	2 (2.0)
Denmark	4 (4.0)
Ireland	7 (6.9)
Mexico	1 (1.0)
South Africa	1 (1.0)
United Kingdom	16 (15.8)
United States	58 (57.4)
*Missing*	8 (7.9)
**Education**	
Associate’s degree	2 (2.0)
Bachelor’s degree	28 (27.7)
Master’s degree	47 (46.5)
Doctoral degree	12 (11.9)
Professional degree (JD, MD)	5 (5.0)
*Missing*	7 (6.9)
**Training specific to delivering** **Arts in healthcare**	
Yes	67 (66.3)
No	27 (26.7)
*Missing*	7 (6.9)

#### Practice Descriptors

Practice descriptors included reporting consistencies and variation in the range and scope of artists’ practices including: (1) who delivers the arts; (2) where and with whom; (3) range of practices and observable changes related to practice.

*Who delivers the arts* included descriptors such as education, training specific to arts in health, current professional role, years of experience, professional collaboration in arts engagement, and the flow of patient referrals. *Where and with whom* included contexts and settings, patient populations, age range of participants, and whether arts engagement took place one-to-one or in a group setting. A range of approaches to practice – or *how* the arts are engaged – described the *range of practices* delivered by artists included the following items: art forms, average number and length of sessions, and whether and how arts engagement was documented. Respondents described public-facing art work created by participants such as performance or exhibition. *Observable changes* included any observable or participant-reported impacts such as change in symptoms, the environment, or participant-verbalized change, as reported by respondents. See Table 3 below for practice descriptors indicating a range of approaches to practice and observable changes.

**TABLE 3:** Practice descriptors.

Who delivers the arts	
**Who delivers the arts with patients** (select all that apply)	
Artists	77 (76.2)
Health professionals	48 (47.5)
Program coordinator or project lead	27 (26.7)
Volunteers	46 (45.5)
Other	5 (5.0)
**Professional collaborations for arts delivery** (select all that apply)	
Artists of same discipline	29 (28.7)
Artists of varying discipline	40 (39.6)
Artists and health professionals	44 (43.6)
Limited to no collaboration	13 (12.9)
Wide range of collaborations	47 (46.5)
**Current place of employment** (Categories)	
Arts organization	16 (15.8)
Hospital/Inpatient palliative care unit	33 (32.7)
University	10 (9.9)
Other: Community, hospice, long-term care/outpatient, self-employed, other	34 (33.7)
*Missing*	8 (7.9)
**Years in current professional role, mean (SD), *n* = 92**	11.26 (9.21)
**Years professionally involved with arts in PC, mean (SD), *n* = 87**	9.99 (8.46)
Range: (0–30)	

**Where and with whom**	

**Settings of arts engagement** (select all that apply)	
Community setting	60 (59.4)
Day hospice	18 (17.8)
Residential hospice	32 (31.7)
Hospital	70 (69.3)
Inpatient palliative care unit	41 (40.6)
In home care	25 (24.8)
Long-term care facility	36 (35.6)
Short-term rehab facility	18 (17.8)
Outpatient treatment center or clinic	41 (40.6)
Other	12 (11.9)
**Common health conditions among patients** (select all that apply)	
Cancer or blood disorders	74 (73.3)
Cardiovascular disease or stroke	55 (54.5)
Dementia	59 (58.4)
Frailty	50 (49.5)
Kidney disease	38 (37.6)
Liver disease	33 (32.7)
Respiratory disease	50 (49.5)
Neurological conditions	58 (57.4)
Chronic pain	51 (50.5)
A wide range of unknown or unspecified health conditions	51 (50.5)
Other	11 (10.9)
**% of time spent with 1:1 or in groups, mean (SD)**	
With individuals on a one-to-one basis, *n* = 92	37.51 (36.77)
With groups of individuals who have a similar health condition, *n* = 92	17.18 (23.98)
With groups of individuals with varying health conditions, *n* = 91	8.00 (18.50)
With groups of individuals who have health conditions and their family members, *n* = 91	14.51 (22.56)
With groups of individuals who have health conditions and health professionals, *n* = 90	6.80 (17.80)
Other, *n* = 94	3.43 (17.70)
**% of patients in the following age ranges, mean (SD), *n*** = **95**	
0–3 years	1.87 (6.44)
4–12 years	5.50 (12.00)
13–18 years	5.22 (10.61)
19–35 years	7.77 (9.74)
36-64 years	25.87 (20.39)
65 years or older	41.49 (31.25)

**Range of practices**	

**Art forms engaged with patients**	
Literary arts	1 (1.0)
Performing arts	26 (25.7)
Visual arts	18 (17.8)
Multidisciplinary or multiple arts forms	48 (47.5)
*Missing*	8 (7.9)
**Average # of arts sessions/wk in the past year, mean (SD), *n*** = **89**	11.89 (10.65)
**Average length of arts session**	
Under 15 min	3 (3.0)
15–30 min	13 (12.9)
30–45 min	13 (12.9)
45 min–1 h	23 (22.8)
More than 1 h	13 (12.9)
Varies by patient population or setting	26 (25.7)
Missing	10 (9.9)
**Average length of arts session (in categories)**	
0–30 min	16 (15.8)
30 min–1 h	36 (35.6)
More than an hour	13 (12.9)
Varies by patient population or setting	26 (25.7)
Missing	10 (9.9)
**% of those who provide patient referrals to the arts, mean (SD)**	
Artist, *n* = 94	8.57 (20.80)
Program coordinator, *n* = 95	9.91 (22.26)
Family members or loved ones, *n* = 94	6.27 (9.50)
Patient self-referral, *n* = 94	12.46 (19.03)
Health professional, *n* = 94	42.55 (37.04)
**Artist documentation of arts sessions** (select all that apply)	
In medical record	24 (23.8)
To share with their supervisors and/or colleagues	49 (48.5)
For professional purposes	57 (56.4)
For research and/or evaluation purposes	51 (50.5)
For other purposes	6 (5.9)
Artist do not formally document arts sessions	11 (10.9)
**Artwork produced shared publicly**	
Yes	61 (60.4)
No	26 (25.7)
*Missing*	14 (13.9)

**Outcomes**	

**Changes observed during or immediately following an arts session** (select all that apply)	
In patient health or symptoms	68 (67.3)
In patient’s environment	21 (20.8)
Verbalized by patient or group	70 (69.3)
Other	24 (23.8)

### Who Delivers the Arts

Responses to current professional role yielded a description of multiple roles played by many survey respondents, such as both program coordinator and artist-in-residence. A wide range of professional collaborations were documented with a prevalence of collaborations occurring between artists and health professionals (44%) followed by artists of varying disciplines (40%) and artists of the same discipline (28%). Referrals were primarily provided by health professionals (43% of referrals) followed by patient self-referral (12%), program coordinator (10%), artist self-referral (9%), and a family member (6%).

### Where and With Whom

Settings and participant or patient populations were described in the findings. Hospitals were the primary setting in which arts engagement was reported among respondents followed by hospice, community settings, outpatient healthcare settings, and finally, long-term care. Significant variation was noted between where respondents were employed and where artists were observed practicing. Respondents reported current employment ranging from hospitals/hospice (33%), arts organizations (16%), self-employment (10%), university settings (10%) and other (12%). That said, respondents reported that artists practiced and collaborate widely in all of the settings listed above and more.

**FIGURE 2:**
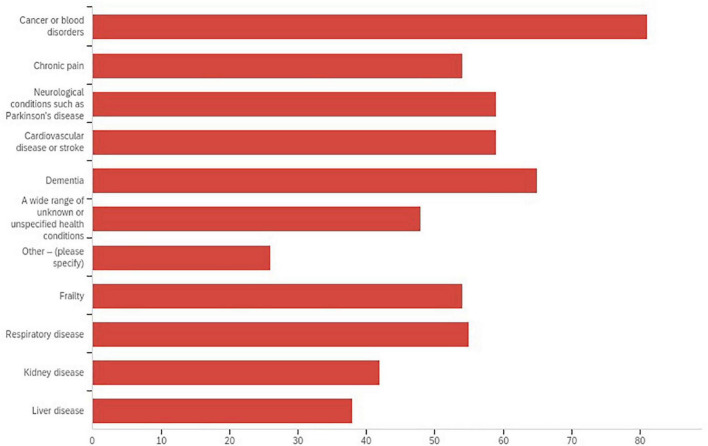
Prevalence of participant populations served.

Cancer and blood disorders were the most prevalent populations served with older adults with dementia reported second. Neurological conditions, frailty, cardiovascular disease and strokes, organ disease and chronic pain were also noted. A majority of respondents (75%) worked with adult populations ages 19 and older with 42% of those working with participants who were 65 years of age and older. See Figure 2 for prevalence of participant populations.

### Range of Practices

Art forms including visual art (18%), literary arts (1%), performing arts (26%) and multidisciplinary arts (48%) were reported and the distribution represented by respondents in the study. An average number and length of sessions, and whether and how arts engagement was documented were reported. Describing practice specifically, an average of 12 sessions were offered per week by a single artist with the majority of sessions lasting between 45 min and an hour. Among respondents, nearly half represented that multidisciplinary, or multiple, art forms (48%) such as music, dance, creative writing and/or visual art were available to patients or service users in their setting.

Professional structures were described including flow of communication, collaboration, documentation, and presentation of public-facing work. Wider variation was described in aims of documentation of arts engagement, however, only 11% of respondents did not document their practice at all, so practice was consistently documented for a range of professional purposes as exemplified by respondent quotations in Table 4. Also, a majority of respondents (60%) publicly exhibited or performed the art work that was created by patients, such as in art exhibition, a gallery space, or a public performance. Approaches to public-facing work such as exhibits, performances and readings of participant-created art work were consistently reported as requiring participant consent as well as discipline-specific artistic proficiencies and resources to execute well. See Table 4 for examples featuring rich descriptions representing a range of contexts and mechanisms at play in artists’ professional practices presenting public-facing work and documenting arts engagement. In this table, the sets of quotations are, respectively, categorized by text exemplifying *what* is occurring, *how* it occurs, and *to what end* under the themes of public-facing work and documentation of practice.

**TABLE 4:** Respondent quotations on public-facing work and documentation of practice*.

	*What?*	*How?*	*To what end?*
*Public-facing, participant-created work*	“Artwork is sometimes shared publicly, usually work by patients with whom we have cultivated a deep artistic relationship, and only with proper record of signed consent” “periodically” “infrequently” “sporadic byproduct” “rarely a driving force” “in the community”	“Consent is essential.” “voluntary” “optional” “Patient directed” “patient led” “collaboratively created for installation” “community partnership”	“making special”, “special occasion”, “celebration” “exhibitions, performances, readings, presentations” “teaching seminars or annual conferences” “fundraisers or grant writing” “for print publication”
*Documentation of practice*	“Artists provide written report to share with team or supervisors.” “Creative arts therapists documents in electronic health record.”	“incident reporting” “personal reflection on arts practice … journaling”	“public relations, media, promotions, social media and fundraising” “funder’s reports, annual reports” “academic research to publish” “program evaluation and tracking”

*The table above presents respondent quotations, which exemplify perceptions of what is occurring, how it occurs, and to what end with regard to artists’ professional practices presenting public-facing, participant-created work and documenting arts engagement.

### Observable Changes

Observable changes were reported numerically as this survey item was designed to select all that apply. These included participant’s health or symptoms (*n* = 68), changes in participant’s immediate environment (*n* = 21), verbalized by participants or group members (*n* = 70), and any other observable change (*n* = 24). Of the respondents, 68 wrote additional comments describing observable changes categorized in analysis as ranging from physical states (i.e., symptom reduction, relaxation, changes in breathing, blood pressure or heart rate as evidenced by monitors, reduced pain medication in one instance), emotional states [i.e., uplift, positive affect, reduced fear or anxiety, well-being scale (WEMWBS) measure improvements in well-being in one instance], mental states (i.e., increased focus, attention, orientation or alertness), and social states (i.e., increased communication, engagement, socialization).

## Thematic Analysis of Benefits and Risks

A total of four open-ended items invited respondents to comment on their perceptions of practice including: (1) Briefly, what does the term *palliative care* mean to you in the context of your professional work?; (2) In your view, what are the primary benefits, if any, of engaging the arts with patients in palliative care?; (3) In your view, what are the primary risks, if any, of engaging the arts with patients in palliative care?; and (4) What else would you like to share about your professional experience engaging the arts in palliative care?

Themes and sub-themes were generated through thematic analysis of codes developed from the qualitative data (Braun and Clarke, 2006, 2014), which centered on perceived benefits and risks of arts engagement with those receiving palliative care and also considered respondents’ definitions of the term *palliative care*. Respondents defined palliative care as “comfort care” and as care for “chronic conditions”, “serious illness” and “care at end-of-life”. Characteristics frequently used to describe palliative care include collaborative, holistic and centered around quality of life using terms such as “comfort care” with “psychosocial” and “spiritual” emphasis. “Meaning making”, “legacy”, “purpose” and “support for family” were also indicated.

For the purposes of this article, three overarching themes were generated focused upon perceived benefits and risks of practice including: (1) enhanced well-being and quality of life of *both* participants and artists; (2) vulnerabilities and safety concerns for *both* participants and artists; and (3) perceived facilitators of and barriers to practice. Respondent quotations and associated themes related to benefits and risks were generated and provided in Table 5.

**TABLE 5:** Themes and sub-themes with respondent quotations.

Perceptions of benefits for *participants*	
**Theme 1a: *Quality of life and well-being of participants due to arts engagement***	

**Subtheme 1:** Physical well-being	“…increased ability to sleep following dance sessions” “reduce symptom burden”

**Subtheme 2:** Mental well-being	“fuller self-expression” “[participant] comments suggesting a redefinition of self as ‘creative”’ “Arts … support the person to continue living and discovering, even in the context of serious illness.” “Art works created by service users are installed in their clinical environment thus making clinical spaces more welcoming and less fearful.”

**Subtheme 3:** Emotional well-being	“expressing the inexpressible” and “You made my day!” “They report gratitude for the opportunity to take their mind to a more positive place.” “expressions of gratitude” “giving families a tangible item is… priceless”

**Subtheme 4:** Social well-being	“increased levels of patients coming together in a circle to participate thus supporting increased levels of social interaction.” “Healthcare professionals experience benefits when art is provided to patients with an end-stage illness. It can improve communication with patients.”

**Perceptions of benefits for *artists***	

**Theme 1b: *Quality of life and well-being of artists due to arts practice located in palliative care***	

**Subtheme 1:** *Artistic growth and personal development*	“Artistically rigorous place to locate one’s practice” “[arts in palliative care] gives great insight into the human condition and I am better equipped to work with more people at this stage of life.”

**Subtheme 2:** *Meaningful employment*	“Satisfying and meaningful employment”; “a deep sense of meaning and purpose” “…rich, varied and valuable” “a very rewarding practice” “…can be life changing” “It’s the most meaningful work I have ever done” “These practices are also hugely rewarding to the practitioner.”

**Subtheme 3:** *Professional collaboration*	“it is important to have artists as part of the multidisciplinary team to work in collaboration with client, carers and other team members for a wholistic approach” “Kindness, gratefulness and open-heartedness of patients, overall” “People – patients, family members, and staff - are very grateful for our service.”

**Perceptions of risks for *participants***	

**Theme 2a: *Vulnerabilities and safety issues for participants or patients***	

**Subtheme 1:** *Art form-specific considerations*	“resident with dementia sampled paintbrush in their mouth.” “Art and music can provoke strong emotions, and the hospital is already a place for emotional landmines.” “Assuming that all music is a universal good to all people all the time is a huge risk … choice, control, music selection, tempo, key, register and volume control [are key].”

**Subtheme 2:** *Adapting for participant population*	“Arts can induce … anxiety in patients that have limited experience, and if this is not … managed correctly by the artist then patients may focus on their short comings” “Inappropriate choice of art form can cause distress to a patient.” “overstimulation. That can cause great restlessness and sometimes anger.” “Overexertion, bringing up too much emotion, frustration or difficulty with materials due to condition”

**Subtheme 3:** *Emotional safety*	“Emotional safety is a priority …” “can bring up painful memories and provoke tears” “patient may feel a social obligation [to say yes] to the artist…” “ensuring that the arts facilitation will not cause a high level of emotional risk and distress for the patient or their families.” “art can de-stabilize and disorganize someone versus stabilizing and fortifying their experience. Collaborate [with health professionals and] creative arts therapists to prevent distress…”

**Perceptions of risks for *artists***	

**Theme 2b: *Vulnerabilities and safety issues of artists***	

**Subtheme 1:** *Training and orientation to healthcare environment or population-specific needs*	“The closer to the bedside, the more training needed.” “not training people to recognize signs of physical distress in patients is a risk” “adapting arts with symptom burden, disease progression, or disability in mind” “vital to be able to assess abilities of participants - physiological changes that prohibit capacities” “…due care must be taken with properly trained staff who understand the impact and power of arts in health.”

**Subtheme 2:** *Awareness among, or collaboration with, healthcare staff*	“A risk is when staff assume that all the arts are good for everyone-No!” “…advocate strongly for collaboration between arts in health and creative arts therapists. There are specific roles that each can play.” “there is some risk of distraction for healthcare providers…” “It is a priority that the artist is integrated into the palliative care process and is given support as required.” “…in United Kingdom generally there is still a great need for education of healthcare professionals about the benefits of arts in health.”

**Subtheme 3:** *Materials or discipline-specific considerations*	“sterilization of [visual art] materials” “follows infection control precautions with arts materials or props “ “with dance, there is a risk of falling” “physical safety [of participants] if they are on pain meds or attending an outpatient class” “music is deeply connected with emotions…”

**Subtheme 4:** *Emotional safety*	“Facilitator burnout, vicarious trauma, and compassion fatigue.” “…both incredibly hard and incredibly rewarding. The care and well-being of the artists that work directly with these patients should be a vital consideration for any organization overseeing arts in palliative care.” “…over involvement, secondary traumatization or burnout because of the existential burden of the work or processes witnessed.” “oversharing by patient or artist” “difficult communications”

**General perceptions of practice**	

**Theme 3: *Facilitators and barriers***	

**Subtheme 1: *Perceived facilitators of practice***	“Person-centered, patient-led and patient-directed” “A **process-based** approach is so effective in achieving a meaningful act or a series of acts that gather meaning. A deeper understanding of **relational autonomy** supports our work. It keeps the person we are supporting at the fore.” “Communication and collaboration with palliative care team, families and participants/patients themselves” “standardize aspects of practice” *(such as Practice Descriptors presented in Table 3)* Training that emphasizes “communication”, “collaboration”, “safety measures such as materials use or fall prevention”, risk assessment and observation skills, facilitation skills, adapting practice for accessibility

**Subtheme 2: *Perceived barriers and limits to practice***	“We. Need. More. Funding. Simple.” “…save healthcare costs in the long-run.” “benefits dramatic, risks minimal, costs modest…” “Short-term nature of engagements due to project funds” “The arts have tremendous value in healthcare. This potential is currently largely untapped. Patients could be FAR happier, relaxed, self-aware with regular arts provision. This would reduce NHS bills into the bargain.” “Potential lack of artist resources, support, supervision, training and development opportunities to address skills stated above” “Emotional expression and emotional safety outlined as a risk for both participants and artists above”

### Perceptions of Benefits of Practice

Respondents identified benefits of the arts as addressing an overarching theme of quality of life followed by subthemes physical mental, emotional, and social well-being. Respondents described “meaning making”, “social engagement”, “symptom relief” and “personal growth and achievement” for participants. Respondents also described artistic growth and development, “rewarding” and “meaningful work”, and social well-being through “professional collaboration” and participant, family and healthcare staff “gratitude” for their work.

### Perceptions of Risks of Practice

With regard to perceived risks, respondents indicated a range of views on vulnerabilities with consideration for participants, artists themselves, and the systems within which artists practice, referenced as the “environment of care” in one instance. Vulnerabilities of participants included examples such as “emotional discomfort of participant”, “emotional safety”, and the term “vulnerability” specifically. Vulnerability of artists featured examples such as “burnout and vicarious or secondary trauma”, “proper training of artists”, value of “collaboration with mental health professionals” and of “navigating environment of care safely”. Notably indicated, though with lesser frequency, was the importance of artists’ assessment and observation skills in order to adeptly navigate the finding that arts engagement can both alleviate anxiety and introduce it. Another finding, however, was that emotions, and even distress, is normal, healthy human experience and as long as health professionals are on hand and working in collaboration, it is natural and even positive for some emotional expression to occur during arts engagement, specifically music.

General perceptions of practice were derived from the final open-ended question and yielded perceived barriers and facilitators as a primary theme. Sub-themes of perceived facilitators of practice included: person-centered and participant-led practice, communication and collaboration, and training and resources for artists. Sub-themes associated with perceived barriers of practice included: resources and funding, lack of support or development opportunities for artists and emotional safety for all involved.

## Discussion

The findings of the study described a prevalence of artists delivering the arts in oncology, and in hospital, hospice, and community settings. A majority of artists have both formal education (92% of artists who responded held a university degree) and additional training in arts in health (60% of artists reported arts in health training). Artists described consistency in practices of professional collaborations, flow of information such as documentation or receiving and making referrals, and public-facing patient-created art work. Variation in practice worth further exploration included adapting specific art forms across a wide range of participants and settings as well as a wide range of administrative structures such as purposes of documentation of practice, and referral sources including health professionals, family members, or family members’ referral. Three overarching themes included: enhanced well-being and quality of life of *both* participants and artists; vulnerabilities and safety concerns for *both* participants and artists; and perceived facilitators of and barriers to practice.

### Significance of Examining “for Whom”

The findings have significance for participants, for artists, for arts in health as a discipline, and for culture, health and well-being sectors, broadly. Critical analysis of patterns of consistency and variation in international artists’ practice advances efforts to recognize, value, and provide infrastructure in support of meaningful and effective interactions during arts engagement for artists and participants alike. Examination of professional perceptions of facilitators and barriers in arts in palliative care informs research and policy aimed at providing access to the arts.

Building upon existing literature, it is valuable to scope key professionals’ perceptions of practice, namely those professionals who have direct experience delivering the arts in palliative care, where outcomes studies rightly focus on patients’ perceptions and self-report (Fancourt and Finn, 2019). Secondly, it is also of use to reach internationally, across palliative and end-of-life care settings, and across patient populations in an effort to document the range and craft a continuum of artists’ professional practices (Anderson et al., 2017; Fancourt and Finn, 2019). Finally, this study offers a survey instrument that may be adapted or built upon to describe artists’ practices and to examine professional perceptions of arts engagement in specific healthcare settings or patient populations.

Key professionals who responded to the survey described benefits to well-being and quality of life for participants based on observation and underpinned by participant self-report. Risks were also reported, such as managing difficult emotions that arise during a session. Emotional expression of participants elicited by arts engagement was met with mixed findings among respondents. This finding is consistent with the literature in which processing emotional expression through arts engagement is frequently associated as an aim and within scope of practice of the creative arts therapies given their mental health education and training. Some respondents cautioned against eliciting emotional expression advocating for additional skills, training, supervision, and in a few instances, creative arts therapies’ credentialing in order to navigate and process difficult emotion, which is consistent with existing recommendations by authors in music and art therapy (Moss, 2016; Van Lith and Spooner, 2018). Others described emotional expression as healthy, normal human experience, and even positive, when navigated in a skilled manner by the artist and re-directed toward the aim of art making. In some instances, an aim of the arts engagement was legacy making or public-facing performance or exhibition, for example. Anxiety was noted to be both correlated with art making, such as when a participant is not confident in their skill or lacks self-esteem, and to be alleviated by art making, such as when the challenge of the art activity is suited to the participant’s skill level. Future research might continue to examine and explore the roles and limits of artists’ practices with consideration for the vulnerabilities present for artists and participants alike in creative processes.

### Significance of Examining “by Whom”

One consistent finding that bears further exploration is the multiple professional roles artists play across patient populations or healthcare settings. In some instances, artists reported working for more than one organization and/or in more than one role such as coordinating arts programs and facilitating arts engagement. This is highlighted by the contrast in findings in “current place of employment” and “settings in which arts engagement occurs”, which points to a divergence of professional pathways for an artist seeking to work in palliative or end-of-life care and leads to questions of a central entry point for an artist seeking to gain employment. A range of respondents described their work in arts in health as merely one aspect of their professional identity regardless of their professional role. For example, some respondents stated that they manage multiple roles and income streams including creating and selling their own artwork professionally whilst also working in other socially-engaged forms of arts practices such as arts education, arts and disability or arts in prison settings.

While artists reported working widely across contexts (i.e., in multiple settings), it was rare that artists were engaged in informing or conducting research, as only four respondents reported doing so in the survey. It may be of use to consider ways to address artists’ professional risks and to support artists’ collective well-being by deepening understanding of the economic, intellectual and cultural components comprising professional work in arts in health broadly, and in arts in palliative care specifically. Artist-led research initiatives are recommended in order to explore these vital elements of practice to ensure workforce well-being and resilience. The findings suggest there is value in considering what skills, structures, resources, and training may best equip artists to promote practices that increase benefits and ameliorate risks for participants, artists, and the wider healthcare and arts organizations and sectors, within which they operate.

An additional finding of significance was the vulnerabilities artists face while working in palliative care. Perceived facilitators and barriers might be more directly explored in future study to better understand and inform these aspects of practice and implement policy to address them. Further, it would be valuable to investigate variables impacting artists such as: job/economic security, training/professional pathways, and organizational structures and resources as relates with workforce resilience.

### Significance of Key Professionals’ Perceptions

Rich description of perceptions of benefits, risks, and overall practice were provided. The volume of written response in open-text items indicated investment in the subject matter by key professionals including artists, health professionals and program coordinators. As demonstrated in the three primary themes, six sub-themes and corresponding respondent quotations in Table 5, participant well-being was impacted in myriad ways and risks outlined that may be ameliorated with specific recommendations made by respondents. Perceived risks in particular indicated future directions for research to inform practice such as training in arts in palliative and end-of-life care, awareness for healthcare staff, art form-specific considerations including adaptations for specific conditions and patient populations and finally, taking steps to ensure the well-being of both the participants and the artists delivering arts engagement.

### Implications

This is the first international survey effort to describe, and further examine, the range and scope of literary, visual, performing and multidisciplinary arts practices delivered by artists with patients in palliative or end-of-life care. The introduction of a survey instrument is a contribution to further conceptualizing mechanisms of practice in an effort to advance the evidence base. Arts in health professional membership organizations were receptive to supporting recruitment for the study, which enhanced the response rate and demonstrated interest in the study topic. As the field of arts in health is evolving, expanding, and professionalizing, it was difficult to ascertain definitively the number of artists working in the clinical subspeciality of palliative care. Given this challenge, the number of respondents (*N* = 101) was significant in itself. Respondents represented international reach, including the four continents of North America, South America, Europe and Africa and thirteen individual nations, even in those instances in which only one response was received from a region such as South Africa, Mexico, or Spain.

### Limitations

Several factors impact the generalizability of findings in the study. The survey was distributed *via* professional membership organizations and networks and therefore had limited reach among those who were not affiliated with formal networks. Further, it was conducted in the English language and therefore limits respondents who do not speak English, as there was not funding to translate the survey for wider reach. These study limitations impacted demographic representation of respondents. More respondents represented the United States and the United Kingdom than other geographical locations represented including Ireland, Australia, Canada, Europe, Mexico, and South Africa. The overall response rate to the survey was not calculated as there was no available data that documented the number of artists working worldwide in palliative and end-of-life care. In the future, it would be beneficial to consider whether forcing responses or amending the tool to fewer items might yield additional data. Finally, while it is beyond the scope of the present study, it is vital to consider the valuable roles of volunteers play in delivering the arts in palliative and end-of-life care settings and is worthwhile to articulate and examine these contributions in their own light.

## Conclusion

Findings demonstrated a wide range of artists’ practices in palliative and end-of-life care, featuring both consistencies in international practice as well as variation. Education and training, key steps in professional preparation, for example, vary widely raising question as to professional pathways for artists. This study highlights a need to further conceptualize and potentially standardize aspects of practice in order to increase uptake of the arts in palliative care and support safe, meaningful and effective implementation for participants and artists alike. Consideration of safe and effective practice for both participants, and equally artists’, well-being is paramount. A primary goal of the survey was to describe key professionals’ perceptions of the range and scope of artists’ practices in the delivery of visual, performing, literary, or multidisciplinary arts with individuals living with life-limiting illness and/or receiving palliative or end-of-life care.

As previously established, evidence supports engaging the arts for health and well-being, pointing to the unique benefits of the arts in palliative care including non-pharmacological symptom management, quality of life and meaning making. An ongoing and international effort to deepen understanding of practice may contribute to an uptake of the arts delivered by artists in palliative and end-of-life care. As practice continues to evolve, clear understanding of aims, approaches, benefits and limits of arts engagement ultimately enhances patients’ experiences of the arts whilst advancing evidenced links between the arts and well-being.

## Data Availability Statement

The raw data supporting the conclusions of this article will be made available by the authors, without undue reservation.

## Ethics Statement

The studies involving human participants were reviewed and approved by University of Florida Institutional Review Board (IRB-02) and Ulster University INHR filter committee. All survey respondents provided their written informed consent to participate in this study.

## Author Contributions

JBL conducted data collection, analysis, and manuscript preparation. SM and LF provided methodological support, manuscript development, and critical appraisal of manuscript. All authors have given the final approval of the manuscript to be submitted for publication.

## Conflict of Interest

The authors declare that the research was conducted in the absence of any commercial or financial relationships that could be construed as a potential conflict of interest.

## Publisher’s Note

All claims expressed in this article are solely those of the authors and do not necessarily represent those of their affiliated organizations, or those of the publisher, the editors and the reviewers. Any product that may be evaluated in this article, or claim that may be made by its manufacturer, is not guaranteed or endorsed by the publisher.

## References

[B1] Anderson K. G. C. Langley J. O’Brien K. Paul S. Graves K. (2017). Examining the artist–patient relationship in palliative care. A thematic analysis of artist reflections on encounters with palliative patients. *Arts Health* 11 67–78. 10.1080/17533015.2017.1413401 31038040 PMC6494112

[B2] Braun V. Clarke V. (2006). Using thematic analysis in psychology. *Qual. Res. Psychol.* 3 77–101. 10.1191/1478088706qp063oa 32100154

[B3] Braun V. Clarke V. (2014). What can “thematic analysis” offer health and wellbeing researchers? *Int. J. Qual. Stud. Health Well-being* 9:26152. 10.3402/qhw.v9.26152 25326092 PMC4201665

[B4] Charmaz K. (2006). Measuring pursuits, marking self: meaning construction in chronic illness. *Int. J. Qual. Stud. Health Well-being* 1 27–37. 10.1080/17482620500534488

[B5] Clift S. (2012). Creative arts as a public health resource: moving from practice-based research to evidence-based practice. *Perspect. Public Health* 132 120–127. 10.1177/1757913912442269 22700576

[B6] Creswell J. W. Creswell J. D. (2017). *Research Design: Qualitative, Quantitative, and Mixed Methods Approaches.* Newbury Park, CA: Sage publications.

[B7] Eva G. Morgan D. (2018). Mapping the scope of occupational therapy practice in palliative care: a European Association for Palliative Care cross-sectional survey. *Palliat. Med.* 32 960–968. 10.1177/0269216318758928 29756556 PMC5946674

[B8] Eysenbach G. (2004). Improving the quality of web surveys: the checklist for reporting results of internet e-surveys (CHERRIES). *J. Med. Internet Res.* 6:e34. 10.2196/jmir.6.3.e34 15471760 PMC1550605

[B9] Fancourt D. Finn S. (2019). What is the Evidence on the Role of the Arts in Improving Health and Well-being? A Scoping Review. Health Evidence Network Synthesis Review. Copenhagen: WHO Regional Office for Europe, 2019. 32091683

[B10] Fancourt D. Ockelford A. Belai A. (2014). The psychoneuroimmunological effects of music: a systematic review and a new model. *Brain Behav. Immun.* 36 15–26. 10.1016/j.bbi.2013.10.014 24157429

[B11] Greer J. A. Applebaum A. J. Jacobsen J. C. Temel J. S. Jackson V. A. (2020). Understanding and addressing the role of coping in palliative care for patients with advanced cancer. *J. Clin. Oncol.* 38 915–925. 10.1200/JCO.19.00013 32023161 PMC7082158

[B12] Henson L. A. Maddocks M. Evans C. Davidson M. Hicks S. Higginson I. J. (2020). Palliative care and the management of common distressing symptoms in advanced cancer: pain, breathlessness, nausea and vomiting, and fatigue. *J. Clin. Oncol.* 38:905. 10.1200/JCO.19.00470 32023162 PMC7082153

[B13] Jensen A. (2014). Considering ‘first, do no harm’ in arts and health practice. *J. Appl. Arts Health* 5 331–339. 10.1386/jaah.5.3.331_1

[B14] Kalton G. (1983). Models in the practice of survey sampling. *Int. Stat. Rev.* 51 175–188.

[B15] Kennett C. E. (2000). Participation in a creative arts project can foster hope in a hospice day centre. *Palliat. Med.* 14 419–425. 10.1191/026921600701536255 11064789

[B16] Knaul F. M. Bhadelia A. Rodriguez N. M. Arreola-Ornelas H. Zimmermann C. (2018). The Lancet Commission on palliative care and pain relief—findings, recommendations, and future directions. *Lancet Glob. Health* 6 S5–S6.

[B17] Lee J. B. McIlfatrick S. Fitzpatrick L. (2021). Arts engagement facilitated by artists with individuals with life-limiting illness: a systematic integrative review of the literature. *Palliat. Med.* 10.1177/02692163211045895 34781774

[B18] Lincoln Y. S. Guba E. G. (1985). *Naturalistic Inquiry.* Thousand Oaks, CA: Sage.

[B19] Lincoln Y. S. Guba E. G. (1986). But is it rigorous? Trustworthiness and authenticity in naturalistic evaluation. *New Dir. Program Eval.* 1986 73–84. 10.1002/ev.1427

[B20] Long C. O. (2011). Cultural and spiritual considerations in palliative care. *Journal of Pediatric Hematology/Oncology* 33 S96–S101. 21952581 10.1097/MPH.0b013e318230daf3

[B21] Macnaughton J. White M. Stacy R. (2005). Researching the benefits of arts in health. *Health Educ.* 105 332–339. 10.1108/09654280510617169

[B22] Meier D. E. Brawley O. W. (2011). Palliative care and the quality of life. *J. Clin. Oncol.* 29 27–50. 10.1200/JCO.2011.35.9729 PMC3139393 21670456

[B23] Miles M. B. Huberman A. M. Saldaña J. (2018). *Qualitative Data Analysis: A Methods Sourcebook.* Thousand Oaks, CA: Sage Publications.

[B24] Mills J. Ramachenderan J. Chapman M. Greenland R. Agar M. (2020). Prioritising workforce wellbeing and resilience: what COVID-19 is reminding us about self-care and staff support. *Palliat. Med.* 34 1137–1139. 10.1177/0269216320947966 32736490

[B25] Morgan D. L. (2007). Paradigms lost and pragmatism regained: methodological implications of combining qualitative and quantitative methods. *J. Mix. Methods Res.* 1 48–76. 10.1177/2345678906292462

[B26] Morgan D. L. (2014). “Pragmatism as a paradigm for mixed methods research,” in *Integrating Qualitative and Quantitative Methods* (Thousand Oaks, CA: SAGE Publications, Inc), 25–44. 10.4135/9781544304533.n2

[B27] Moss H. (2016). Arts and health: a new paradigm. *Voices: A World Forum for Music Therapy.* 16. 10.1177/0898010105282465 16449751

[B28] Moseholm E. Fetters M. D. (2017). Conceptual models to guide integration during analysis in convergent mixed methods studies. *Methodol. Innov.* 10:2059799117703118.

[B29] Moss H. O’Neill D. (2009). What training do artists need to work in healthcare settings? *Med. Humanit.* 35 101–105. 10.1136/jmh.2009.001792 23674706

[B30] Moss H. O’Neill D. (2014). Aesthetic deprivation in clinical settings. *Lancet* 383 1032–1033. 10.1016/s0140-6736(14)60507-9 24665475

[B31] Moss H. O’Neill D. (2019). Narratives of health and illness: arts-based research capturing the lived experience of dementia. *Dementia* 18 2008–2017. 10.1177/1471301217736163 29022362

[B32] National Organization for Arts in Health (2017). *Arts, Health, and Well-Being in America.* San Diego, CA: NOAH.

[B33] Peng C. S. Baxter K. Lally K. M. (2019). Music intervention as a tool in improving patient experience in palliative care. *Am. J. Hosp. Palliat. Care* 36 45–49. 10.1177/1049909118788643 30045627

[B34] Raw A. Lewis S. Russell A. Macnaughton J. (2012). A hole in the heart: confronting the drive for evidence-based impact research in arts and health. *Arts Health* 4 97–108. 10.1080/17533015.2011.619991 24244217 PMC3827737

[B35] Ruel E. Wagner W. E. III Gillespie B. J. (2015). *The Practice of Survey Research.* Thousand Oaks, CA: Sage.

[B36] Sepúlveda C. Marlin A. Yoshida T. Ullrich A. (2002). Palliative care: the World Health Organization’s global perspective. *J. Pain Symptom Manage.* 24 91–96. 10.1016/s0885-3924(02)00440-2 12231124

[B37] Shannon-Baker P. (2016). Making paradigms meaningful in mixed methods research. *J. Mix. Methods Res.* 10 319–334. 10.1177/1558689815575861

[B38] Sonke J. (2015). “Professionalizing the arts in healthcare field,” in *Managing Arts Programs in Healthcare*, ed. Lambert P. D. (New York, NY: Routledge), 50–62. 10.4324/9781315754420-13

[B39] Sonke J. Helgemo M. Pesata V. L. (2019). Arts in health mapping project: Florida. *Arts Health* 11 264–271. 10.1080/17533015.2018.1494451 31038437

[B40] Sonke J. Pesata V. Lee J. B. Graham-Pole J. (2017). Nurse perceptions of artists as collaborators in interprofessional care teams. *Healthcare* 5:50. 10.3390/healthcare5030050 28867778 PMC5618178

[B41] Sonke J. Lee J. B. Helgemo M. Rollins J. Carytsas F. Imus S. (2018). Arts in health: considering language from an educational perspective in the United States. *Arts Health* 10 151–164. 10.1093/her/16.6.671 11780707

[B42] Staricoff R. L. (2004). *Arts in Health: A Review of the Medical Literature.* London: Arts Council England.

[B43] Tan M. K. B. (2018). Connecting reminiscence, art making and cultural heritage: a pilot art-for-dementia care programme. *J. Appl. Arts Health* 9 25–36. 10.1386/jaah.9.1.25_1

[B44] Tan M. K. B. (2020). Towards a caring practice: reflections on the processes and components of arts-health practice. *Arts Health* 12 80–97. 10.1080/17533015.2018.1494452 31038438

[B45] Van Lith T. Spooner H. (2018). Art therapy and arts in health: Identifying shared values but different goals using a framework analysis. *Art Ther.* 35 88–93. 10.1080/07421656.2018.1483161

[B46] Wilson C. Bungay H. Munn-Giddings C. Boyce M. (2016). Healthcare professionals’ perceptions of the value and impact of the arts in healthcare settings: a critical review of the literature. *Int. J. Nurs. Stud.* 56 90–101. 10.1016/j.ijnurstu.2015.11.003 26696399

[B47] World Health Organization (2018). *What is Palliative Care.* Geneva: WHO.

